# Topical Treatment with Cord Blood Serum in Glaucoma Patients: A Preliminary Report

**DOI:** 10.1155/2018/2381296

**Published:** 2018-07-25

**Authors:** Emilio Campos, Piera Versura, Giuseppe Giannaccare, Adriana Terzi, Silvia Bisti, Stefano Di Marco, Marina Buzzi

**Affiliations:** ^1^Ophthalmology Unit, DIMES, Alma Mater Studiorum, University of Bologna, Bologna, Italy; ^2^Emilia Romagna Cord Blood Bank-Transfusion Service, S.Orsola-Malpighi Teaching Hospital, Bologna, Italy; ^3^Vision Lab, DISCAB, University of L'Aquila, L'Aquila, Italy

## Abstract

**Purpose:**

To report data which happened to be observed in two glaucoma patients treated with Cord Blood Serum (CBS) eye drops.

**Design:**

A case report and retrospective data analysis.

**Methods:**

CBS topical eye drops, characterized in advance for growth factors (GFs) content, were administered for two months with the aim to relieve their subjective symptoms, in two patients who had referred ocular surface discomfort, although in absence of any sign of keratopathy. As patients were also affected by advanced glaucoma at risk of vision loss and under treatment with hypotensive drugs, they had been also monitored over the same period with IOP controls and visual field tests in our unit.

**Results:**

During subsequent visits, data from Mean Deviation and Pattern Standard Deviation in the visual fields were retrospectively collected and compared with before and after treatment with CBS, and an amelioration was observed.

**Conclusions:**

CBS contains a combination of GFs, which potentially exert a neuroprotective action and elect CBS as an interesting natural source to be delivered in neurodegenerative ocular disorders. The incidentally observed amelioration in these two patients deserves further investigation in this respect.

## 1. Introduction

The term glaucoma identifies a group of ocular multifactorial pathologies with the characteristic triad of the intraocular pressure (IOP) increase, degenerative phenomena affecting the optic nerve head, and a progressive deterioration of the visual field. Some forms of glaucomatous neuropathy are however diagnosed without clinical evidence of ocular hypertension, and also progressive visual loss in glaucoma may occur even with pharmaceutical controlled IOP values.

The disease is now increasingly seen as one of the central nervous system age-related neurodegenerations [1, 2]. Taking into account both the presence of normotensive glaucoma and the recent observations on the presence of neurotoxic waste in glaucomatous subjects which might induce downstream events like oxidative stress [3], glial activation [4], neurotrophin deprivation [5], and mitochondrial dysfunction [6, 7] it seems possible to hypothesize that glaucoma is a multifactorial neurodegenerative disease where the main outcome is represented by ganglion cells death [2]. Accordingly it is becoming a relevant issue to test neuroprotective agents in patients with glaucoma to slow down or even block the progression of the disease [8, 9].

In the present paper incidentally observed data are reported on two patients who had been treated with Cord Blood Serum (CBS) eye drops prescribed with the aim to relieve their subjective symptoms of surface discomfort.

It is well known that the cord blood is rich with trophic factors whose content might vary according to several unpredictable parameters probably during gestation and labor [10, 11]. Recently CBS has been used as eye drops for pathologies related to cornea but the possibility that such a mixture of factors might be useful even in retinal disease exists and deserves to be tested [12, 13].

Preliminary results from these cases provide evidence of a positive outcome of experimental hypothesis, as CBS eye drop treatment efficiently ameliorates visual field in glaucomatous patients.

### 1.1. Treatment

Cord Blood Serum (CBS) eye drops had been prepared as previously described in detail [12] for epithelial healing purpose. Briefly, the umbilical cord blood (UCB) was obtained from mothers with vaginal or cesarean section delivery after informed consent, and all steps were performed according to standard operating procedures and guidelines edited by the Foundation for the Accreditation of Hematopoietic Cellular Therapy (FACT). For the purpose of preparing eye drops, blood samples were collected from ex utero placenta vessels and were clotted for 2 hours at room temperature, centrifuged, and frozen at -80°C for quarantine period. After this period sera were thawed, pooled, diluted by 20% in sterile PBS, filtered, finally aliquoted into COL-20 medical device (Biomed, Modena, Italy) in single dose one-day vials, packed, frozen, and stored at -80°C.

One aliquot in each lot had been retained for further testing of selected growth factors (GFs), in particular IL-10, IL-13, basic FGF, PDGF-bb, b-NGF, EGF, and TGF-*α*. Samples were evaluated by using commercially available multiplex bead-based sandwich immunoassay kits (Bio-Rad Laboratories, CA, USA), by means of the Bio-Plex Protein Array System (Bio-Rad Laboratories, CA, USA) as detailed elsewhere [14].

### 1.2. Case Presentation

The setting of this study was the Ophthalmic Unit at S.Orsola-Malpighi Teaching Hospital, Alma Mater, Studiorum University of Bologna, where the treatment with CBS topical eye drops is a therapeutic option in patients suffering from dry eye associated with severe subjective symptoms of discomfort [12]. In the period of March-September 2016, two patients who had referred ocular surface discomfort unresponsive to previous therapy, although in absence of any sign of keratopathy, were administered with CBS topical eye drops for a total of two months. As patients were also affected by POAG and under treatment with hypotensive drugs, they had been also monitored over the same period with IOP controls and visual field tests in our unit. Data from the retrospective analyses of these examinations and of the back history of these patients is here described.

### 1.3. Case 1

Patient n. 1 was a 66-year old male, suffering from diabetes type I, requiring insulin therapy since 2006. The patient also presented associated diabetic retinopathy, and a primary open angle glaucoma (POAG) had been diagnosed in January 2014. Values of IOP were successfully maintained within normal range with hypotensive topical drugs. In January 2016, the patient had referred to increasing irritating symptoms of eye discomfort, described as burning, itchiness, and feeling sand in his eyes, scored as OSDI (Ocular Surface Disease Index) [15] of 65 out of 100, with a VAS (Visual Analogue Score) [16] score of pain of 74 mm out of 100 and no difference between eyes. Slit lamp evaluation had not shown epithelial damage, also with the aid of fluorescein vital stain observed with the blue cobalt filter, and there were no signs of inflammation, but only a reduced Tear Film Break-Up Time (TFBUT) of 7 seconds in both eyes had been recorded. A therapy with hyaluronic acid (HA) based tear substitute to be administered 4 times/day in both eyes was prescribed.

At a subsequent visit in April 2016, the patient reported no relief from severe symptoms (OSDI: score 62 out of 100, VAS: 80 out of 100 mm) which remained severe, despite the regular administration of HA, increased from 4 to multiple times each day. As recorded from the history of the patient's charts, previous therapy with anti-inflammatory drugs had turned to be unsuccessful, and in May 2016 the patient was proposed to receive a treatment with topical CBS, as a compassionate unconventional therapy. The rationale for this therapy was to reduce the pain symptoms for which the previous therapeutic attempts had turned to be unsuccessful.

The patient signed the informed consent, specifically designed for this purpose, and started administration of the CBS eye drops in June 2016, with the posology of 0.4 ml (8 drops) in each eye, each day for a total of two months. In Table 1 the GF dosages determined for the two CBS lots were administered during the first and second month of treatment.

In September 2016, the patient reported a significant relief from subjective symptoms of discomfort. OSDI score was reduced: 24 out of 100 and VAS was also reduced: 35 mm out of 100. During this visit, the IOP resulted in the normal range, and the analyses of the visual field tests performed since 2014 were carried out.

**Figure 1:**
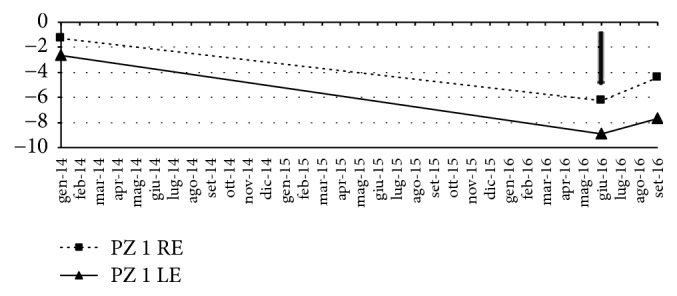
Patient 1. Trend of the mean deviation (MD) from January 2014 to September 2016. The arrow indicates the beginning of treatment with CBS eye drop. RE: right eye; LE: left eye.

**Figure 2:**
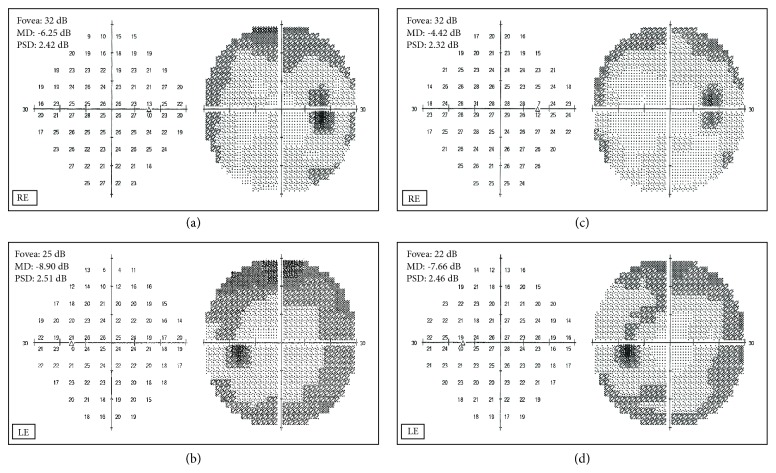
Patient 1. Central 30-2 visual field test recorded in June 2016 (a, b) and September 2016 (c,d), in right (RE) and left (LE) eye.

In Figure 1 the mean deviation (MD) values recorded in January 2014, June 2016, and September 2016 were graphed. A significant lowering in MD values was observed which was followed by an amelioration in correspondence with the period of treatment with CBS, in the figure highlighted with the arrow. In Figure 2 the central 30-2 visual field tests before ((a) right eye RE; (b) left eye LE) and after (c, d) the CBS treatment were shown. An amelioration of the defect was observed in all the four quadrants in both eyes, as it is demonstrated by the unvaried Pattern Standard Deviation (PSD) values.

### 1.4. Case 2

Patient n. 2 was a 60-year old female, diagnosed with suffering from POAG for ten years, under treatment with hypotensive drugs and regularly controlled IOP twice a year which appeared maintained within normal ranges. During a control visit in January 2016, the patient had reported irritating symptoms of eye discomfort, described as burning, itchiness, and feeling sand, mainly in her right eye (RE), over the last six months. The situation had been managed with the use of several types of tear substitutes, none of them successful in symptom relief. Symptoms were scored in the RE as OSDI of 74 out of 100 and a VAS score of pain of 85 mm out of 100. In the left eye (LE) the symptoms were defined by the patients as light and acceptable: OSDI score was 22 out of 100 and VAS 21 mm out of 100. Slit lamp evaluation had not shown epithelial damage, and there were no signs of inflammation, but only a reduced Tear Film Break-Up Time (TFBUT) of 4 seconds in RE and 8 seconds in LE had been recorded. A therapy with hyaluronic acid (HA) based tear substitute to be administered 4 times/day in both eyes was prescribed.

In March 2016, the patient was proposed to receive in her RE a treatment with topical CBS, as a compassionate unconventional therapy, with the aim to reduce the pain symptoms for which the previous therapeutic attempts had turned to be unsuccessful. The therapy for the contralateral LE was maintained with HA based tear substitutes.

The patient signed the specifically designed informed consent and started administration of the CBS eye drops in April 2016, with the posology of 0.4 ml (8 drops) in RE, each day for a total of two months. In Table 2 the GF dosages determined for the two CBS lots were administered during the first and second month.

On September 2016, the patient reported a significant relief from subjective symptoms of discomfort; in RE the OSDI was 28 out of 100, with VAS: 32 mm out of 100. Also in LE a reduction was observed, with OSDI score determined as 16 out of 100 and VAS 15 mm out of 100. During this visit, also the IOP was measured, which resulted in the normal range in both eyes and the analyses of the visual field tests performed since 2006.

**Figure 3:**
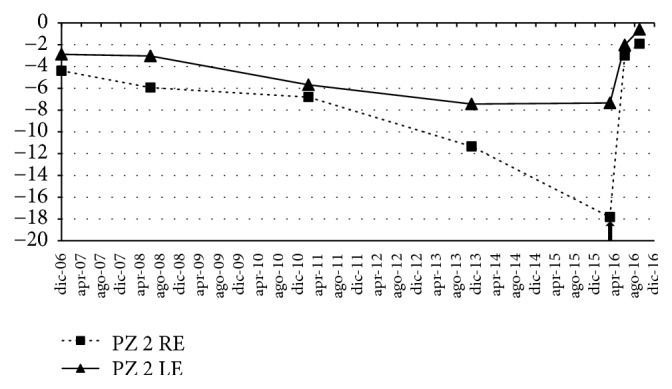
Ten-year trend of the mean deviation (MD), from December 2006 to September 2016. The arrow indicates the beginning of treatment with CBS eye drop. RE: right eye; LE: left eye.

In Figure 3 the mean deviation (MD) values recorded over several visits performed from December 2006 through September 2016 were graphed. A significant progressive lowering in MD values was observed which was followed by an important amelioration in correspondence with the period of treatment with CBS, in the figure highlighted with the arrow. It has to be noted that the increase in MD values was recorded either in the treated right eye or in the untreated left eye. Moreover, the MD values recorded in September 2016, four months after the end of the CBS eye drop administration, showed in both eyes a further amelioration.

**Figure 4:**
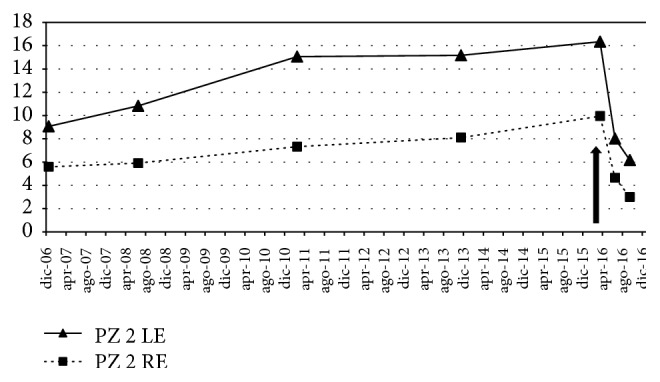
Ten-year trend of the Pattern Standard Deviation (PSD), from December 2006 to September 2016. The arrow indicates the beginning of treatment with CBS eye drop. RE: right eye; LE: left eye.

The improvement is also demonstrated by the analysis of the PSD shown in Figure 4. A progressive worsening in PSD values had been observed over ten years, whereas a rapid change was recorded in correspondence with the CBS eye drop treatment, either in the treated right eye or in the untreated left eye.

**Figure 5:**
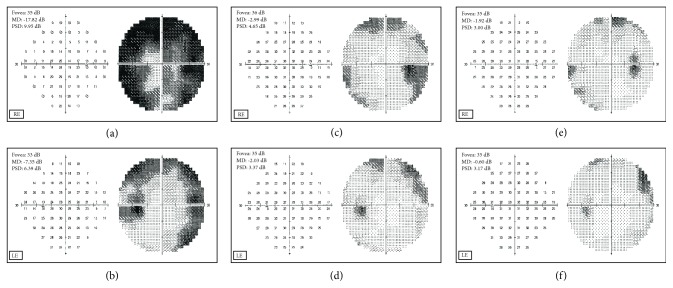
Patient 2. Central 30-2 visual field test recorded in March 2016 (a, b) before the start of CBS treatment, June 2016 (c,d) at the end of CBS treatment, and September 2016 (e, f) after four months from the end of CBS treatment, in right (RE) and left (LE) eye.

In Figure 5 the central 30-2 visual field tests before ((a): right eye RE; (b): left eye LE), at the end (c, d) and after four months (e, f) from the end of the CBS eye drop treatment were shown. An amelioration of the defect was observed in all the four quadrants in both the treated RE and the untreated LE.

Taking together these observations, a positive effect also in the left untreated eye could be recorded, which suggests a neural cross-talk mechanism between the eyes.

## 2. Discussion

Neuroprotective treatment in degenerative ophthalmological diseases is currently proposed by the administration of natural products such as Crocins from saffron [17] or specific synthetized molecules such as Memantine [18], Citicoline [19], Brimonidine [20, 21], Homotaurine [22], and Polyphenols [23, 24]. Clinical studies related to neuroprotection in ocular diseases are at an early stage and although promising it is difficult to figure out whether neuroprotective approach will have useful clinical developments in the near future. In fact, unfortunately, almost all clinical trials of neuroprotection in ophthalmologic disease have failed to show efficacy so far, despite encouraging preclinical studies [25, 26].

This report demonstrated that topical CBS eye drops administered for two months in two patients suffering from glaucoma determined an amelioration of parameters from visual field test, as compared to before and after the treatment. Among neuroprotective agents to be tested, the possibility of using growth factors from cord blood arises from a variety of observation in preclinical and clinical studies, for instance, in amyotrophic lateral sclerosis [27]. We had determined in the CBS eye drops the levels of GFs involved in promoting corneal epithelial healing, but some of them had also been shown to exert neuroprotection mechanism in experimental models of glaucoma, such as TGF-*α* [28], b-NGF [29], and PDGF-bb [30]. The report only allows us to speculate that these GFs, potentially in a synergic action with others not tested specifically on this occasion, may have played a role in the amelioration of the visual field parameters. Another speculation relates to the amelioration effect in the contralateral untreated eye of patient n.2. The possibility arises that the bilateral response might be a consequence of cross-talk between eyes which could be further discussed. We considered our findings from these two patients a serendipity with high potential to be expanded on a more numerous and prospective basis.

## Figures and Tables

**Table 1:** Levels of growth factors contained in the CBS eye drops administered to patient n.1, for the first and second month of treatment, respectively. EGF: epidermal growth factor; TGF-*α*: transforming growth factor; b-NGF: basic nerve growth factor; PDGF-bb: platelet-derived growth factor; b-FGF: basic fibroblast growth factor; and IL: interleukin.

	**month**	**EGF**	**TGF-** **α**	**b-NGF**	**PDGF-bb** **∗**	**b-FGF**	**IL-10**	**IL-13**
**Lot n.1**	first	152.7	15.7	2.0	1.99	104.2	1.8	41.4

**Lot n.2**	second	148.8	12.8	1.7	1.75	76.9	1.5	25.0

Case n.1: values expressed in pg/ml sample, except for *∗*= ng/ml.

**Table 2:** Levels of growth factors contained in the CBS eye drops administered to patient n.2, for the first and second month of treatment, respectively. EGF: epidermal growth factor; TGF-*α*: transforming growth factor; b-NGF: basic neural growth factor; PDGF-bb: Platelet-derived growth factor; b-FGF: basic fibroblast growth factor; and IL: interleukin.

	**months**	**EGF**	**TGF-** **α**	**b-NGF**	**PDGF-bb** **∗**	**b-FGF**	**IL-10**	**IL-13**
**Lot n.3**	first	232.5	19.3	1.4	1.94	96.4	2.9	62.4

**Lot n.4**	second	242.6	19.8	1.2	2.43	44.3	1.2	27.9

Case n. 2: values expressed in pg/ml sample, except for *∗*= ng/ml.
